# Physical Activity and Social Support to Promote a Health-Promoting Lifestyle in Older Adults: An Intervention Study

**DOI:** 10.3390/ijerph192114382

**Published:** 2022-11-03

**Authors:** Wei-Yang Huang, Hsuan Huang, Cheng-En Wu

**Affiliations:** 1 Physical Education, National Taiwan College of Performing Arts, Taipei 11464, Taiwan; 2 Department of Occupational Therapy, National Cheng Kung University, Tainan 701401, Taiwan; 3 Office of Physical Education, Tamkang University, No. 151, Ying-Zhuan Rd., Tamshui, New Taipei City 251301, Taiwan

**Keywords:** super-aged society, physical activity, social support, health-promoting lifestyle, pedometer

## Abstract

The purpose of this study was to understand the effect of intervention in the form of physical activity and social support while co-exercising to promote a health-promoting lifestyle in older adults. This study openly recruited 60 healthy male older adults, all of whom wore a wrist-worn Garmin device walker. Experimental group A was monitored by a special person and walked together as a group, experimental group B walked independently outdoors, and the control group went about their daily routine as normal. All participants recorded daily steps and calorie consumption data for eight weeks. The results of the study showed that when walking together in experimental group A, the number of daily walks and calories consumed could be maintained at a high level due to the effect of group and social support. The number of daily walks in experimental group B was inconsistent due to the lack of motivation to walk on their own. The control group consumed the lowest number of calories in their daily routine. The results show that physical activity and social support have positive explanatory power for a health-promoting lifestyle. Finally, physical activity in older adults should be promoted, and social support for interaction with peers can effectively promote a healthy lifestyle and respond to the arrival of the super-aged society in advance.

## 1. Introduction

Taiwan will become a super-aged society by 2025 when about 20% of the population will be over the age of 65. In other words, one out of every four or five people will be an older adult [1]. As the birth rate continues to decline, more than half of Taiwan’s population is expected to be aged 50 or above by 2034. This demographic development has widespread ramifications for Taiwan’s National Health Insurance (NHI) system. A super-aged society will put severe strains on the healthcare system, making disease prevention and proactive healthcare imperative in a changing Taiwan. Prevention is better than cure, and health-promoting lifestyles are important for older adults and are also a global issue of common concern [2]. There is now a greater awareness of preventive healthcare services and healthy lifestyles in older adults for early prevention or the reduction of chronic diseases and their complications [3]. Therefore, understanding health-promoting lifestyles in older adults is of great significance.

Edington et al.’s research points out that the pursuit of a good quality of life requires health promotion [4]. Health promotion is not only a preventive behavior for diseases and health problems but also has the characteristics of positive growth and actualizing tendencies. It does not only involve behaviors that are meant to prevent diseases or avoid health problems [5]. The WHO defines health-promoting as enabling people to increase control over their own health that covers a broad range of social and environmental interventions designed to benefit and protect the health and quality of life of individuals by addressing and preventing the causes of ill health [6]. Health-promoting is not a specific prevention of diseases or health problems. It is a kind of approach to prevention; that is, self-actualization-oriented behavior that guides individuals to maintain or improve their health and actively establish new positive behaviors [7,8,9,10]. According to the U.S. Department of Health and Human Services, unhealthy behaviors and lifestyles are identified as one of the leading causes of death [11]. However, a health-promoting lifestyle refers to lifestyle improvements that lead to satisfaction, success at work, and physical health and consists of the following six dimensions: physical activity, nutrition, health responsibility, spiritual growth, interpersonal support, and stress management [12,13,14]. Scholars point out that health-promoting means any act that is aimed at strengthening a person’s physical, mental, and social well-being by ensuring that a person’s behavior, social conditions, and environment are conducive to having good health, a firm mental condition, a long life, and a good quality of life [2]. An active lifestyle is generally considered to be a health-promoting behavior [15].

Due to demographic changes, it will become more important for older adults to maintain high levels of physical activity [16]. Many studies have pointed out that there are complex reasons for the lack of PA in older adults. Among these many reasons, “no mentor” and “no peers” are the most common, and many older adults do not know what to do when it comes to physical activity [17,18,19,20].

It is generally believed that walking is the most suitable physical activity for older adults, and the quantification of walking can be expressed in steps or distance, in addition to walking time. As for the intensity of walking activities, it can be expressed in speed or stride frequency [21]. Many studies have confirmed that the use of pedometers can accurately monitor walking behavior and can promote walking tools [22,23]. Therefore, exploring the effect of physical activity on health promotion in older adults forms one of the motivations for this study. In addition, many older adults lack companionship, experience loneliness, and spend more time engaged in sedentary behaviors during the day and night [24]. In particular, the lack of social support for older adults leads to a lonely life, which may lead to cognitive decline, depression, heart disease, and other related health problems [25,26]. Social support for older adults refers to sources of primary influences on health-promoting behavior, such as family (parents or siblings), peers, and providers of healthcare (nurses and doctors). Therefore, the impact of social support in older adults on health promotion is also one of the motivations for this study.

Based on the above motivations, this study proposes to improve the health and lifestyle of older adults through an intervention involving physical activity and social support during common exercise. We attempt to solve the following three problems: 1. What were the physical activities (number of walks) and health-promoting lifestyles among older adults? 2. What is the status of social support and health-promotion lifestyles among older adults? 3. How do physical activity (number of walks) and social support in older adults promote health-promoting lifestyles? According to the research question, the following research hypotheses are proposed. Hypothesis one: walking contributes to a healthy lifestyle in older adults. Hypothesis two: Social support helps older adults to maintain healthy lifestyles. Hypothesis three: Physical activity and social support can effectively promote health-promoting lifestyles in older adults.

## 2. Materials and Methods

### 2.1. Research Participants

This study used the elderly at the Taipei Senior Learning Center as the research population and openly recruited 60 healthy male older adults. The sample size was in line with Gay’s proposal that the total sample size for comparative studies should be at least 60 people [27]. All participants were able to make decisions according to their own will (we excluded those unable to participate due to their age, intelligence, physical condition, personal circumstances, identity, or social or economic conditions). The participants first filled in their personal background information, including their age, weight, height, previous occupational status, and current residential status. The participants were randomly assigned to one of three groups of 20 people: experimental group A (EGa; average age 70.8 ± 6.2 years), experimental group B (EGb; average age 71.1 ± 4.5 years), or the control group (EGc; average age 71.5 ± 5.9 years). There were no significant differences in the groups’ age, height, and weight, indicating that the results of this random allocation of participants were homogeneous, as is shown in Table 1. All participants in this study signed an informed consent form according to their personal wishes, in line with scientific and ethical principles (contents include: non-smokers, no orthopedic disease or heart disease, can perform daily life without help, usually do not participate in an exercise program, willing to abstain from taking supplements that increase muscle mass or sarcopenia during the study). During the 8-week experiment, the control group went about their daily routines as usual. This study was approved by the Jen-Ai Medical Foundation Dali Jen-Ai Hospital: Human Body Research Ethics Committee, approval number 110–96.

### 2.2. Research Materials

The eight-week experimental methods of the three groups are shown in Table 2. The physical activity intervention of the participants in this study was walking, using a wrist-worn Garmin device (trade name: vívosmart HR fitness bracelet), using vibrations during walking to count steps, and electronic computing products through the 3D accelerator records in the chip. Items that can be measured include steps, time, walking distance, calorie consumption, fat burning, physical activity, etc. The Garmin device can store 4 weeks of data [28,29,30]. Participants in this study were able to assess the results of their steps daily or per weekend. They could see the distance they walked and their calorie expenditure through a daily memory setting. They removed the pedometer before bedtime, and they gathered once a week to integrate the data from the Garmin devices.

### 2.3. Testing Method

#### 2.3.1. Garmin Built-In Steps and Calories

Garmin’s vívosmart HR is a smart wearable bracelet with a built-in pedometer sensor and barometric altimeter (to sense whether the wearer is climbing or climbing a ladder), which can distinguish the difference in energy consumption on flat ground or when climbing steps and can automatically count the number of steps taken, distance traveled, and calories burned. The data are synchronized with a mobile phone. After 12:00 every night, all vívosmart records are reset to zero and recalculated, but the previous records are automatically stored and retained. Vívosmart HR provides the Android and iOS dual-system support app “Garmin Connect”. You can connect a vívosmart HR to mobile devices via Bluetooth to change its settings and stream data. This advanced vívosmart HR sports smart bracelet is easy to operate and versatile. It has automatic calculation, strong stability, and good accuracy. The amount of daily walking of older adults is related to their level of physical activity. Tudor-Locke et al. studied the classification criteria of daily walks in older adults, including sedentary life (<2500 steps/day), low activity (2500–5000 steps/day), somewhat active (5000–8000 steps/day), active (8000–11,000 steps/day), and highly active (>11,000 steps/day) [31].

#### 2.3.2. Health-Promoting Lifestyle Profile Scale of Older Adults

This study revises the “Revision and Testing of the Chinese Version of the Health-Promoting Lifestyle Profile” by the Taiwanese scholar Chen et al. in 1997 [32]. The first draft of the scale was completed, and after expert content validity, 150 older adults from the Taipei Senior Learning Center were randomly selected as pre-test participants. The total cumulative explained variance of the pre-test completed scale reached 63.71%, which had construct validity. A reliability analysis was conducted in order to understand the consistency and stability of the scale. The Cronbach’s α value was used to analyze the internal consistency of the items with the same factors. The Cronbach’s α value of the reliability analysis results was between 0.74 and 0.86. The internal consistency of this questionnaire is quite high. A factor analysis extracted six factors, including health responsibility (5 items), physical activity (6 items), nutrition (7 items), spiritual growth (5 items), social support (6 items), and stress management (5 items), for a total of 34 items named the “Health-Promoting Lifestyle Profile Scale of Older Adults” (Table 3). This questionnaire uses a four-point Likert scale with the following responses: 1 = strongly disagree, 2 = slightly disagree, 3 = slightly agree, and 4 = strongly agree [33]. The scores ranged from 2.5 to 4 on average, with higher scores indicating a participant’s more frequent or regular participation in a health-promoting lifestyle. According to the large amount of background information on the elderly in the literature, this study selected background variables that are easier for the elderly to answer and avoided personal health questions. We included age, height, weight, previous occupation status, and current living status [34,35,36,37].

In this study, the physical activity and social support of older adults were used as independent variables. Physical activity was calculated by calculating the number of steps and calories per day. Social support was calculated for the walking group (experimental group A). Both the above tests pass the Health-Promoting Lifestyle Profile Scale. The average scores were interpreted as: 4.00–3.50 is strongly agree, 3.49–3.00 is agree, 2.99–2.50 is somewhat disagree, 2.49–2.00 is disagree, and below 1.99 is strongly disagree.

### 2.4. Control Variable

This study used healthy older male adults as participants, so older female adults and older adults with disabilities were not within the scope of this study. In addition, Garmin’s vívosmart HR device was operated by a research person at a consultation point, and participants aggregated data from the Garmin devices once a week.

### 2.5. Statistical Analysis

Descriptive statistics for daily walking steps, calorie consumption data, and Health-Promoting Lifestyle Scale are presented as mean ± standard deviation. Shapiro-Wilk tests were performed on all dependent variables for the three sets of data to check the assumption of normality, and the results showed that all data supported the assumption of a normal distribution. One-way ANOVA between the three groups was used for multiple comparisons and the LSD post hoc test (applicable to many-to-one comparison as the number of tests is small and the significance level is small) was used with the overall significance level set to *p* < 0.05. Multiple regression analysis was used to measure the explanatory power of older adults’ physical activity and social support for health-promoting lifestyles in older adults. The statistical analyses were performed using SPSS 20.0 software (IBM^®^, Armonk, NY, USA).

## 3. Results

### 3.1. Analysis of Background Variables for All Participants

Background variables were collected for the 60 participants who were recruited for this study, in addition to data on their age, weight, and height. The previous occupational status and current residential status of all participants were also investigated. According to the previous occupational statistics of the participants, 9% retired from cultural and educational institutions, 8% from public utilities, 7% from the hospitality industry, 8% from the transportation industry, 7% from the construction industry, 7% from the manufacturing industry, 8% from the news and advertising industry, 7% from the healthcare industry, 12% from the entertainment industry, 15% from the services industry, and 14% from the general business industry, showing that these older male adults have a wide range of previous occupations. Since the recruited participants all live in cities, they were all retired from their former workplaces. Their current living situations showed that 5.7% live alone, 59.0% live with their spouse, 33.8% live with their spouse and children, and 1.5% live with friends. This shows that living with a spouse is the most common, followed by living with a spouse and children, which together account for 92.8% of the total sample.

### 3.2. Analysis of Daily Walking, Distance, and Calorie Consumption

The average stride length of the participants in the three groups was 60.35 cm to 61.14 cm. From Monday to Sunday after the 8th week of the experiment, the average number of steps per day and the average daily calorie consumption were measured using the F test and the LSD post hoc test, as shown in Table 4. The results of the study found no significant difference in the mean stride length’s F-test results. The average daily steps and calorie consumption of experimental group A on weekdays (Monday to Friday) were better than those of experimental group B and the control group. Experimental group A and experimental group B were better than the control group in terms of mean steps and calorie consumption on weekends (Saturday and Sunday). The above shows that the daily life of the control group was sedentary, the average number of steps per day was less than 2500 steps, and the calorie consumption was less than 200 kcal. In addition, when comparing experimental group A with experimental group B, it is possible that EGa achieved this number of steps by engaging in group activities while being monitored by special personnel on weekdays. Experimental group B may lack the driving force to walk on their own, and the amount of daily walking was inconsistent. We found that the average number of steps per day was lower on weekends than on weekdays, and all three groups of participants consumed fewer calories on weekends than on weekdays, suggesting that older adults may have family gatherings, home breaks, or travel or leisure activities on weekends. Finally, it was found that the average weekly steps and calorie consumption of experimental group A were left-biased, indicating that experimental group A exhibited a group effect when they were monitored by a dedicated person. The number of steps went from “somewhat active (5000–8000 steps/day)” to “active (8000–11,000 steps/day)” on weekdays and back to “somewhat active” on Fridays. We thus confirm hypothesis one: The physical activity of walking contributes to a healthy lifestyle in older adults.

### 3.3. Analysis of the Health-Promoting Lifestyle Profile Scale

After the end of the experiment, all participants were analyzed using the “Health Promotion Lifestyle Scale for Older Adults”, as is shown in Table 5. From the six subscales, both experimental group A and experimental group B had the highest average score for physical activity, followed by social support. Conversely, the lowest average comes from physical activity for the control group, followed by the social support of the control group. In the overall scale, the highest average score was experimental group A, followed by experimental group B, and the control group was the worst by a significant margin (F = 6.752, *p* < 0.05). From the six subscales, the most significant difference among the three groups was physical activity, followed by social support, as is shown in Table 6. The abovementioned data show that both experimental group A and experimental group B engaged in more physical activity in the form of walking. After eight weeks, the subscale of the Health Promotion Lifestyle Scale showed strong agreement with physical activity, followed by social support. In addition, when comparing experimental group A with experimental group B, the biggest difference in the six factors was social support. EGa engaged in group activities with a special person monitoring on weekdays, which meant that they went on many walks. Experimental group B’s walks were inconsistent in terms of the number of daily walks, as they may have lacked the motivation to exercise with friends or peers on their own. Social support was thus shown to have a positive effect on health-promoting lifestyles in older adults. We thus confirm hypothesis two: Social support helps older adults to promote healthy lifestyles.

### 3.4. Evaluating Health-Promoting Lifestyles in the Older Adults

First, we explored the explanatory power of older adults’ physical activity on the health-promoting lifestyle questionnaire, defined here as multiple regression model 1, as shown in Table 7 and Table 8. The independent variables were health responsibility, nutrition, spiritual growth, social support, and stress management and the dependent variable was physical activity. The overall explanatory power of the independent variable (physical activity) was 74.5% (adjusted R^2^ = 0.729 (72.9%), standard error of estimate = 0.616, change in R^2^ = 0.745, change in F value = 17.643, *p* < 0.001). The overall multiple regression model reached a significant level, and the independent variables were tested afterward (health responsibility β = 0.408, nutrition β = 0.429, spiritual growth β = 0.417, social support β = 0.623, and stress management β = 0.436). These results indicate that the health-promoting lifestyle scale correlated fairly well with physical activity. That is, the number of walks in this study could positively explain the five factors of the health-promoting lifestyle scale.

Next, we looked at the explanatory power of the older adults’ social support on the health-promoting lifestyle questionnaire, defined here as multiple regression model 2, as shown in Table 9 and Table 10. The independent variables were health responsibility, physical activity, nutrition, spiritual growth, and stress management and the dependent variable was social support. The overall explanatory power of all independent variables for social support was 66.3% (adjusted R^2^ = 0.648 (64.8%), and the overall regression model was significant, F = 8.173, *p* < 0.001). The overall multiple regression model reached a significant level, and the independent variables were tested afterward (health responsibility β = 0.426, physical activity β = 0.539, nutrition β = 0.477, spiritual growth β = 0.413, and stress management β = 0.465). This indicates that the health-promoting lifestyle scale correlated well with social support. That is to say, social support in this study could effectively promote a health-promoting lifestyle.

Based on the above multiple regression analysis, after eight weeks of wearing wrist-worn Garmin devices in older male adults, we found that physical activity and social support had a positive effect on health-promoting lifestyles. These results confirm the acceptance of hypothesis three: Physical activity and social support can effectively promote health-promoting lifestyles in older adults.

## 4. Discussion

In this study, the use of pedometer intervention to monitor the number of daily walks and calorie consumption in the participants did indeed improve the health-promoting lifestyle of older adults. After eight weeks of pedometer intervention, the difference between participants was statistically significant (*p* < 0.05), which is consistent with the results of many other studies [38,39,40,41]. After eight weeks of group walking with monitoring and guidance by special personnel, the average daily amount of walking in experimental group A showed an inverted U shape. The data showed that the average number of steps per day was 8253, which equates to an active physical activity (PA) level, and the factors of health promotion and lifestyle can effectively explain the amount of walking (R^2^ = 0.745), showing that the number of walks had a 74.5% effect on the health-promoting lifestyle of older adults. This study provides strong evidence that regular PA was a very effective way to promote health [42]. Golinowska et al. also confirmed that a healthy lifestyle can improve the overall physical and mental health of older adults [2]. Conversely, excessive calorie intake, insufficient exercise, sedentary TV watching, and low physical activity are listed as among the unhealthy lifestyle habits of older adults [43,44]. The average daily number of steps in the control group was 2479 (<2500 steps/day). These people lived a sedentary life, which causes a rapid decline in muscle mass and other body systems, which is harmful to their health. Some scholars have studied the effect of the amount of steps per day on body tissue, including Swartz et al. Their study reported that individuals who achieved ≥5000 steps/day had a significantly lower prevalence of adverse cardiometabolic health indicators [45]. Shimizu et al.’s study shows that individuals who achieve ≥7000 steps/day have better immune systems [46]. Research by Mitsui et al. shows that reaching 8000 steps/day can effectively improve older adults’ BMI and help them to avoid metabolic syndrome [47]. A study by Swartz et al. reported that individuals who achieved ≥5000 steps/day had a significantly lower prevalence of adverse cardiometabolic health indicators [45]. Another point worth exploring is that the weekend exercise or physical activity of the EG was an autonomous behavior, meaning that it cannot be controlled. The physiological changes caused by holidays may have caused interference factors in this study. This is a confounding factor, and Tsirtsakis has shown that regular exercisers on weekdays are more effective at improving cognitive performance than those who exercise only on weekends [48]. However, Haase confirmed that moderate-to-vigorous physical activity, whether spread out during the week or concentrated on the weekend, does not differ significantly in terms of its health benefits [49].

In addition, experimental group A was the walking group and experimental group B was the autonomous walking group, and the biggest difference between the two was the guidance of companions and special personnel. Experimental group A had a fixed time and place, and it was easy to produce a peer effect. The factor of having a health-promotion lifestyle can effectively explain social support (R^2^ = 0.663), showing that social support has a 66.3% effect on the health-promotion lifestyle of older adults. A study by Dobarrio-Sanz et al. confirmed that social support, such as exercising with friends, sharing sports experiences with friends, and obtaining support from friends, can promote health [50]. A study by Rovniak et al. confirms that social support plays an important role in increasing physical activity [51]. Victor et al. show that social support increases participation in physical activities [52]. The participants in this study were most likely to live with their spouses, followed by their spouses and children. These two living arrangements accounted for 92.8% of participants. This shows that older adults live with their families, can be cared for by family members, have companions when exercising, and can strengthen social relationships, which is consistent with the findings of many other scholars [34,53]. Numerous studies have confirmed that more emotional support from others means that individuals gain greater enjoyment from PA and also makes people more motivated to engage in PA or exercise [54,55,56,57]. A study by Booth et al. showed that having active friends was a significant predictor of PA behavior [58]. Research by Lindsay Smith et al. confirmed that social support was particularly important for increasing PA in older adults [34]. A study by Langhammer et al. confirmed that those with social support, family support, or friends or peers who exercise with them are more likely to continue exercising than those who do not receive support [36]. A comparison of three groups found that social support for PA was particularly important for older adults who do not exercise regularly [57]. This may be why many report that social support is particularly effective at increasing the activity levels of inactive older adults [34]. To sum up, these findings suggest that social support is important for increasing healthy behaviors and may be especially important for sedentary older adults or individuals who may need additional motivation to participate in PA [6,59,60]. This study uses physical activity intervention measures for older adults, but the EG was also divided into group walking and autonomous walking. The purpose of these groupings was to understand the differences between groups that contain peers, friends, and other social support structures.

The health-promoting lifestyle scale for older adults contains physical activity (duration, frequency, intensity), spiritual growth (sleep, positive growth, life goals, vision), social support (friends, peers), stress management (satisfaction with oneself), and exercise (decompression, full of challenges, avoid fatigue) factors. These have the potential to promote health through physical activity and social support, and older adults have different levels of interpersonal relationships. Higher levels can optimize health, and the results of this study are consistent with those of many other scholars [61,62,63,64]. Our regression analysis found that walking as a physical activity and social support had a highly positive predictive effect on health-promoting lifestyles in older adults, and we found that physical activity was positively correlated with social support. That is, having a partner to exercise with can improve the willingness of the elderly to exercise [34]. This view is consistent with the findings of research by Yamada et al., who concluded that older adults should be provided with adequate guidance and social support to promote physical activity [65]. The main barriers to social support in older adults may be long-term loneliness (lack of spouse, friends, peers, and family), lack of interaction with family members or friends, and an inability to obtain information about health from the surrounding environment [66]. Secondly, there are barriers to the use of electronic technologies in daily life by older adults. Mobile phone and tablet technologies are complex, and, compared with the younger generation, older adults lack social interaction and communication skills [67]. Therefore, exercise, social, and internet technologies can help to promote the physical and mental health of older adults [13].

This study has several limitations. First, the study sample was a convenience sample of older adults in the community. Furthermore, recruiting from communities in only one metropolitan area in Taiwan may limit the generalizability of the results to other rural areas or countries. Second, this study mainly focused on physical activity and social support to conduct a cross-sectional study of health-promoting lifestyles in older adults and could therefore not fully include all six factors of health promotion.

## 5. Conclusions

Our data show that PA and social support have a positive effect on the health and lifestyle of older adults. The use of dedicated personnel to guide and control the number of daily walks for older adults, and the use of group walking sessions to build peer-to-peer interpersonal relationships, can help older adults achieve a health-promoting lifestyle. Large-scale longitudinal studies are needed in the future to determine the causal relationship between the six major factors of health-promoting lifestyles in older adults, which may help provide public health recommendations for health promotion in older adults.

## Figures and Tables

**Table 1:** Background characteristics of the participants in the cross-sectional study.

Variable	EGa (n = 20) M ± SD	EGb (n = 20) M ± SD	CG (n = 20) M ± SD	*F* Value	*p*-Value
Age (years)	70.8 ± 6.2	71.1 ± 4.5	71.5 ± 5.9	0.74	0.247
Height (cm)	170.7 ± 6.5	171.4 ± 7.2	169.8 ± 6.5	0.92	0.175
Weight (kg)	75.4 ± 6.3	77.1 ± 6.9	76.2 ± 7.1	1.13	0.106

EGa means experimental group A, EGb means experimental group B, CG means control group, and M ± SD means mean ± standard deviation.

**Table 2:** The experimental methods of the three groups.

Group	Method	Content
EGa	Wrist-worn Garmin device walker	It was stipulated that from Monday to Friday, from 4:00 to 5:30 pm, they would gather at regular and fixed points (group activities, special personnel monitoring) and walk together in the park for at least 30 min. The participants were advised to engage in more outdoor activities on Saturdays and Sundays. They recorded their total steps and calorie consumption each day.
EGb	Wrist-worn Garmin device walker	It was stipulated that independent outdoor walking was required from Monday to Friday, and participants were recommended to engage in more outdoor activities on Saturday and Sunday. They recorded their total steps and calorie consumption each day.
CG	Wrist-worn Garmin device walker	The participants went about their daily routines. They recorded their total steps and calorie consumption each day.

EGa means experimental group A, EGb means experimental group B, and CG means control group.

**Table 3:** Health-Promoting Lifestyle Profile Scale of Older Adults.

**Factor 1: Health Responsibility**	
1	I watch TV programs about improving health.
2	I discuss my health concerns with a health professional.
3	I get a second opinion when I suspect advice given by my healthcare professional.
4	I examine my body at least once a month to detect any physical changes.
5	I attend educational programs on healthcare.
**Factor 2: Physical activity**	
6	I exercise vigorously for 30 min or more at least three times a week (e.g., brisk walking, climbing stairs).
7	I participate in physical activity at a mild to moderate level (e.g., walking continuously for 30–40 min five times or more per week).
8	I do stretching exercises at least three times a week.
9	I participate in leisure physical activities (such as swimming pool, spa, dancing, tai chi, and croquet).
10	I exercise while doing daily activities (such as using stairs instead of elevators, walking short distances, cleaning, and shopping).
11	I check my pulse rate while exercising.
**Factor 3: Nutrition**	
12	I choose a diet low in fat, saturated fat, and cholesterol.
13	I limit my intake of sugar and foods that contain sugar (sweets).
14	I eat 1.5 to 4 bowls of staple food, including cereals, rice, and noodles, daily.
15	I eat two to four fist-sized pieces of fruit daily.
16	I eat three to five plates of vegetables daily.
17	I drink 350 to 500 mL of dairy daily.
18	I eat 115–300 g of meat, chicken, fish, dried beans, eggs, or beans daily.
**Factor 4: Spiritual growth**	
19	I get enough sleep.
20	I feel I am growing and changing in a positive way.
21	I believe that my life has a purpose.
22	I look forward to the future.
23	I concentrate on pleasant thoughts before bed.
**Factor 5: Social support**	
24	I discuss exercise patterns with my friends.
25	I compliment my friends’ athleticism.
26	I exercise with my friends.
27	I show concern for my friends.
28	Playing sports with my friends makes me happy.
29	I get support from friends while exercising.
**Factor 6: Stress management**	
30	I accept things I cannot change in my life.
31	I feel satisfied and calm with myself.
32	Exercise makes me less stressed.
33	I find that every day is exciting and challenging.
34	I calm myself to avoid fatigue.

Average score scale: 4.00–3.50 is strongly agree, 3.49–3.00 is agree, 2.99–2.50 is somewhat disagree, 2.49–2.00 is disagree, and below 1.99 is strongly disagree.

**Table 4:** Descriptive statistics. One-way ANOVA, and LSD post hoc test of average stride, steps, and calories among the three groups.

Day	EGa (n = 20)	EGb (n = 20)	CG (n = 20)	*F*-Value	*p*-Value	Post Hoc LSD
	M ± SD	M ± SD	M ± SD			
Stride, cm	60.35 ± 5.57	61.14 ± 6.13	60.67 ± 5.36	0.317	>0.642	-
Monday, steps	7857.25 ± 103.41	6203.36 ± 141.37	2445.03 ± 234.63	5.125 *	<0.005	EGa > EGb > CG
Calories, kcal	392.86 ± 15.26	310.15 ± 12.83	121.25 ± 7.61	5.336 *	<0.003	EGa > EGb > CG
Tuesday, steps	8436.47 ± 164.55	5795.42 ± 157.66	2547.17 ± 219.48	8.192 *	<0.0007	EGa > EGb > CG
Calories, kcal	421.82 ± 21.83	289.77 ± 13.72	126.36 ± 8.43	7.461 *	<0.001	EGa > EGb > CG
Wednesday, steps	9365.71 ± 231.32	6121.75 ± 104.29	2458.15 ± 212.48	13.857 *	<0.0001	EGa > EGb > CG
Calories, kcal	468.29 ± 32.57	306.09 ± 15.65	120.91 ± 7.46	10.743 *	<0.0004	EGa > EGb > CG
Thursday, steps	8079.34 ± 135.46	5335.92 ± 128.62	2483.24 ± 198.72	7.928 *	<0.0008	EGa > EGb > CG
Calories, kcal	403.97 ± 18.41	266.80 ± 16.85	122.16 ± 7.89	6.592 *	<0.002	EGa > EGb > CG
Friday, steps	7527.69 ± 117.51	5242.91 ± 136.47	2462.53 ± 175.64	4.973 *	<0.007	EGa > EGb > CG
Calories, kcal	376.35 ± 16.62	262.15 ± 12.91	122.13 ± 7.67	4.657 *	<0.008	EGa > EGb > CG
Saturday, steps	4516.23 ± 1819.57	4472.46 ± 1753.74	3173.46 ± 1547.62	3.579 *	<0.012	EGa = EGb > CG
Calories, kcal	225.81 ± 9.36	223.62 ± 8.79	158.67 ± 6.15	3.354 *	<0.032	EGa = EGb > CG
Sunday, steps	4544.57 ± 1916.18	4529.81 ± 1887.93	3094.53 ± 1676.31	3.471 *	<0.026	EGa = EGb > CG
Calories, kcal	227.23 ± 8.53	226.49 ± 9.13	154.73 ± 5.34	3.228 *	<0.043	EGa = EGb > CG

* *p* < 0.05. EGa means experimental group A, EGb means experimental group B, CG means the control group, and M ± SD means mean ± standard deviation. Stride refers to the distance of each step when walking, also known as “step distance”; stride = distance/steps. LSD: Post hoc comparisons, of which Fisher’s least significant difference is abbreviated.

**Table 5:** The lowest, highest, and mean scores of different aspects of the Health-Promoting Lifestyle Profile Scale.

Group	Health Responsibility	Physical Activity	Nutrition	Spiritual Growth	Social Support	Stress Management
EGa mean	3.09 ± 0.41	3.84 ± 0.33	3.28 ± 0.45	3.11 ± 0.38	3.76 ± 0.42	3.65 ± 0.46
minimum score	13	19	18	15	21	16
maximum score	18	24	25	19	24	19
EGb mean	3.06 ± 0.43	3.47 ± 0.27	3.21 ± 0.33	3.09 ± 0.44	3.18 ± 0.37	3.14 ± 0.45
minimum score	14	15	19	16	18	13
maximum score	17	23	26	19	23	18
CG mean	3.05 ± 0.47	2.26 ± 0.42	3.20 ± 0.38	3.01 ± 0.41	2.87 ± 0.38	3.06 ± 0.47
minimum score	12	10	17	13	14	12
maximum score	18	18	25	16	20	17

EGa means experimental group A, EGb means experimental group B, and CG means control group.

**Table 6:** Descriptive statistics. One-way ANOVA and LSD post hoc test for the Health-Promoting Lifestyle Profile Scale.

Group	EGa (n = 20)	EGb (n = 20)	CG (n = 2 0)	*F*-Value	*p*-Value	Post Hoc LSD
	M ± SD	M ± SD	M ± SD			
Health responsibility	3.09 ± 0.41	3.06 ± 0.43	3.05 ± 0.47	0.511	>0.462	-
Physical activity	3.84 ± 0.33	3.47 ± 0.27	2.26 ± 0.42	9.326 *	<0.001	EGa > EGb > CG
Nutrition	3.28 ± 0.45	3.21 ± 0.33	3.20 ± 0.38	0.715	>0.317	-
Spiritual growth	3.11 ± 0.38	3.09 ± 0.44	3.01 ± 0.41	3.226 *	<0.015	EGa = EGb > CG
Social support	3.76 ± 0.42	3.18 ± 0.37	2.87 ± 0.38	6.184 *	<0.003	EGa > EGb > CG
Stress management	3.65 ± 0.46	3.14 ± 0.45	3.06 ± 0.47	5.491 *	<0.006	EGa > EGb > CG
Total scale	3.46 ± 0.41	3.21 ± 0.44	2.91 ± 0.43	6.752 *	<0.002	EGa > EGb> CG

* *p* < 0.05. EGa means experimental group A, EGb means experimental group B, CG means control group, and M ± SD means mean ± standard deviation. LSD: Post hoc comparisons, of which Fisher’s least significant difference is abbreviated.

**Table 7:** Summary of the step volumes of the EGa for multiple regression model 1.

Model	R	R^2^	Adjusted R^2^	Estimated Standard Error	R^2^ Change	*F* Change	*p*-Value
1	0.863 (a)	0.745	0.729	0.616	0.745	17.643 *	<0.001

* *p* < 0.05. (a) refers to the predictor variables of this study, including constant, health responsibility, nutrition, spiritual growth, social support, stress management, etc.

**Table 8:** Physical activity coefficients of EGa for multiple regression model 1.

Model		Unstandardized Coefficients		Standardized Coefficients	t	*p*-Value
		B	Std. Error	Beta		
1	(Constant)	7.226	0.529	-	8.114 *	<0.001
	Health responsibility	0.257	0.027	0.408	3.545 *	<0.004
	Physical activity	0.431	0.001	0.731	8.237 *	<0.001
	Nutrition	0.292	0.022	0.429	4.831 *	<0.002
	Spiritual growth	0.296	0.024	0.417	4.429 *	<0.003
	Social support	0.374	0.005	0.623	7.346 *	<0.001
	Stress management	0.305	0.016	0.436	5.182 *	<0.001
	Physical activity	0.431	0.001	0.731	8.237 *	<0.001

* *p* < 0.05.

**Table 9:** Summary of the social support of the EGa for multiple regression model 2.

Model	R	R^2^	Adjusted R^2^	Estimated Standard Error	R^2^ Change	*F* Change	*p*-Value
2	0.814 (a)	0.663	0.648	0.023	0.663	8.173 *	<0.001

* p < 0.05. (a) refers to the predictor variables of this study, including constant, health responsibility, physical activity, nutrition, spiritual growth, stress management, etc. EGa means experimental group A.

**Table 10:** Social support coefficients of EGa for multiple regression model 2.

Model		Unstandardized Coefficients		Standardized Coefficients	t	*p*-Value
		B	Std. Error	Beta		
2	(Constant)	4.591	0.231	-	5.696 *	<0.001
	Health responsibility	0.237	0.012	0.426	4.464 *	<0.007
	Physical activity	0.278	0.001	0.539	5.187 *	<0.001
	Nutrition	0.269	0.008	0.477	4.823 *	<0.003
	Spiritual growth	0.225	0.017	0.413	3.812 *	<0.012
	Stress management	0.253	0.010	0.465	4.636 *	<0.007
	Social support	0.436	0.001	0.568	5.911 *	<0.001

* *p* < 0.05. EGa means experimental group A.

## Data Availability

The experimental results used real data obtained from the study participants. The measurement data were obtained after the training program. The participants agreed with the data structure via a confirmation, and this confirmation can be disclosed with reasonable availability. All of the datasets on which the conclusions of the paper rely are available to editors, reviewers, and readers.
